# iBehavior - A Smartphone-Based Ecological Momentary Assessment Tool for the Assessment of Behavior Change in Neurodevelopmental Disorders

**DOI:** 10.21203/rs.3.rs-2787281/v1

**Published:** 2023-04-17

**Authors:** Andrew Dakopolos, Dana Glassman, Haleigh Scott, Michael Bass, David Hessl

**Affiliations:** University of California, Davis; University of California, Davis; University of California, Davis; Northwestern University; University of California, Davis

## Abstract

The purpose of the present study was to describe the content and function of *iBehavior*, a smartphone-based caregiver-report electronic ecological momentary assessment (eEMA) tool developed to assess and track behavior change in people with intellectual and developmental disabilities (IDDs), and to examine its preliminary validity. Ten parents of children (ages of 5–17 years) with IDDs (n = 7 with fragile X syndrome; n = 3 with Down syndrome) rated their child’s behavior (aggression and irritability, avoidant and fearful behavior, restricted and repetitive behavior and interests, and social initiation) using *iBehavior* once daily for 14 days. At the conclusion of the 14-day observation period, parents completed traditional rating scales as validation measures, as well as a user feedback survey. Parent ratings using *iBehavior* showed emerging evidence of convergent validity among domains with traditional rating scales including the Behavior Rating Inventory of Executive Function 2 (BRIEF-2), Aberrant Behavior Checklist – Community (ABC-C), and Conners 3. iBehavior was feasible in our sample, and parent feedback indicated high overall satisfaction. Results of the present pilot study indicate successful implementation and preliminary feasibility and validity of an eEMA tool for use as a behavioral outcome measure in IDDs.

## Introduction

Efforts to reliably measure behavior, internal states, cognition, and experiences of people with intellectual and developmental disabilities (IDDs) possess methodological challenges. Although approaches for self-report among individuals with IDDs are emerging^1–4^, barriers related to communication, insight, and cognitive functioning persist. Thus, traditional retrospective proxy-report questionnaires are still predominantly utilized to characterize behavior^5–7^, as diagnostic supplements^8–10^, and in clinical trials as primary outcomes^11,12^ for those with IDDs.

When applied rigorously, proxy approaches are psychometrically supported, well-validated, and cost-effective^13^. Despite these benefits, proxy-reported questionnaires can produce rater estimates of behavior prone to systematic biases^14^. Retrospective reporting has been shown to be susceptible to recall bias^15^, and can be influenced by factors such as the salience or outcome of the behavior^16^, as well as the rater’s mental or emotional state at the time of rating^17,18^. Raters may also over- or under-estimate their ratings, particularly in those with IDDs^19^.

Electronic ecological momentary assessment (eEMA) is a measurement method with characteristics that may improve the validity and reliability of reporting on behaviors commonly associated with IDDs. eEMAs encompass various techniques including diary recordings, experience sampling (i.e., multiple, randomly sampled time-points for observation or report throughout the day), and mobile- or web-based applications^20–22^. eEMAs enable raters to directly report an individual’s behavior in near-real-time, across contexts, within flexible periods of time to establish an individual’s “typical behavior” based on both fluctuations and aggregates of all behavior recorded during the observation period^22^.

eEMA methods are newly emerging in the field of IDDs. A recent study by Wilson and colleagues demonstrated feasibility and reliability of a self-report experience sampling (i.e., external events, internal states, and emotions) eEMA measure piloted with 19 adults with mild to moderate intellectual disability^21^. Participants received individualized training on how to use the eEMA mobile app, and practiced completing all items with study personnel and a caregiver before commencing their 7-day trial period. Participants completed on average 33.8% of their ratings. Split-half comparisons indicated internal reliability; however, items were not validated against other established constructs or measures^21^.

In another set of studies, Ness and colleagues evaluated the Janssen Autism Knowledge Engine (JAKE) for use as an outcome measure in clinical trials with 29 youth diagnosed with autism^23^. Parents rated a subset of customizable questions derived from the Autism Behavior Inventory (ABI) twice-weekly, allowing for an analysis of day-to-day fluctuations in autism-specific behaviors^24^. However, compliance/use rates, feasibility, and reliability of the eEMA “daily tracker” were not reported^24^.

In the present pilot, proof-of-concept study, we sought to describe the content and function of iBehavior, a caregiver-report eEMA tool developed by our team, to assess and track behavior in people with IDDs, and to examine its preliminary validity focusing on 10 families and a select set of behavior domains across a 14-day observation period. Given the small sample size, we elected to wait to report reliability statistics until more data is available.

## Method

All procedures performed in this study involving human participants were in accordance with the ethical standards of the institutional and/or national research committee and with the 1964 Helsinki Declaration and its later amendments or comparable ethical standards. The study was approved by the Institutional Review Board at University of California, Davis (No.1865834).

**The** iBehavior **mobile application**. *iBehavior* is a smartphone-based (iPhone or Android) eEMA app designed for use by caregivers to assess maladaptive and prosocial behaviors of people with IDDs, with a focus on its future application as an outcome measure for clinical trials. *iBehavior* content and function development was informed by a panel of stakeholders (parents, teachers, clinicians, researchers, and an FDA representative; see Acknowledgments) invested in the assessment and treatment of children with IDDs, and by a Delphi study focused on Down syndrome, fragile X syndrome, and autism. The Delphi panel agreed on six domains to include in iBehavior (see Table 1).

The *iBehavior* app was built using Nativescript, a framework for creating native mobile applications that targets multiple platforms (i.e., android and iOS). Data collected through the *iBehavior* app is encrypted and securely transmitted in real time to REDCap (Research Electronic Data Capture; Harris et al., 2009, 2019), which for this study is hosted and managed by the UC Davis Clinical and Translational Science Center (CTSC). REDCap database linkage is established via a study code which links their device to a REDcap database using REDCap’s application programming interfaces (APIs).

No personal identifying information (PHI) is recorded within the app or transmitted from the user’s device to REDCap. *iBehavior* content (domain items, language, scaling) can be individually added and/or modified in real time and pushed to the app for users. This feature enables the study team to field test content and respond to feedback in a timely manner. *iBehavior* also includes automatic, configurable notifications sent via SMS text messaging to remind users to begin their observations, and when to complete ratings.

**Figure 1:**
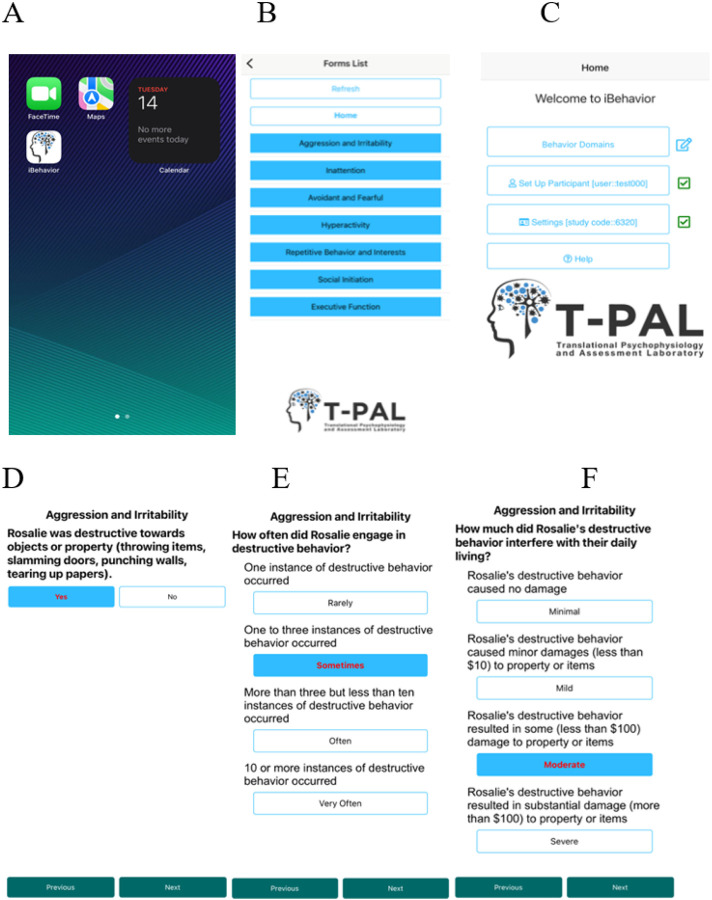
iBehavior mobile application Screenshots of the iBehavior application on an iPhone. (A) Selection of the App on the home screen. (B) Home page of the app and selection of the Behavior Domains. (C) Selection of the specific behavior domain the user is rating (Aggression and Irritability). (D) Initial Yes or No answer selection indicating whether the behavior occurred during the observation period. (E) Rating the frequency of the behavior, with anchoring terms. (F) Rating the intensity of the behavior, with anchoring terms.

Currently, each *iBehavior* domain includes 6–8 items representing discrete types of behavior. For each behavior, the user is prompted to answer a yes/no question about whether the behavior occurred during the observation period. If “yes,” the app prompts the user to record the frequency (“rarely,” “sometimes,” “often,” or “very often”) and intensity (i.e., the degree of interference with daily functioning; “minimal,” “mild,” “moderate,” or “severe”) of the behavior. A key strength is that anchoring text accompanies, and is specific to each behavioral item, and provides specific information to guide a reliable rating (e.g., number of instances of the behavior; duration; degree of interference or distress). See Fig. 1.

In addition to specific behavior domains, iBehavior also includes situational questions, which are always presented before ratings are made. The situational questions include the total time (in hours) the user observed their child, the primary location of observation, whether the child was physically sick, and two questions regarding the child’s quality of sleep the night prior.

### Participants.

Participants included 10 parents of children with IDD. Informed consent was obtained from all individual participants included in the study. All users identified as biological mothers, and nine were married. All parents graduated from high school, and seven earned a bachelor’s degree or higher. The children (2 females, 8 males) were between the ages of 8 to 17 years and were diagnosed with Down syndrome (DS; n = 3) and fragile X syndrome (FXS; n = 7). All children had intellectual disability, with nine previously documented by our study team using the Stanford-Binet 5th Edition (SB5), with full scale IQ deviation scores ranging from 31 to 68 (m = 57.1, sd = 14.1) and significant delays in adaptive behavior.

### Procedure.

**Figure 2:**
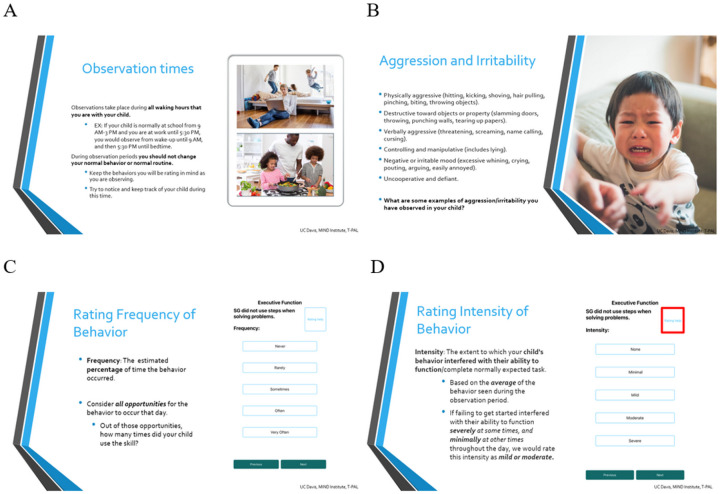
iBehavior user training. Select slides from iBehavior user training. (A) Introduces observation times and clarifies when the caregiver is responsible for recording behavioral data. (B) Discusses domain specific behaviors in relation to the participant’s child. (C and D) calibrates the user to intensity and frequency ratings respectively.

To begin, caregivers received one-on-one iBehavior training with a trained clinician via videoconference. Training lasted approximately one hour and consisted of three components. First, parents were guided to install the app, and set up their child as a participant. Next, parents were trained on the structure of the app, how eEMAs are conducted, and how to approach frequency and intensity ratings of behaviors. Parent users also specified two times they preferred to receive text message reminders to begin and end daily observations (generally shortly after waking up and late in the evening after all contact with their child). Finally, parents participated in a calibration interview, in which behaviors across each domain were explored. Parents provided examples of their child’s behaviors they thought aligned with behaviors presented in the app, and were encouraged to ask questions and elaborate on their responses. If parents identified behaviors that did not align with those in the domain, the trainer redirected interpretation of their child’s behavior and discussed specific areas of misalignment and scoring. See Fig. 2.

After training, parents completed 14 days of iBehavior observational ratings in the following domains: (1) Irritable Mood and Aggression, (2) Avoidant, Fearful and Nervous Behavior, (3) Stereotyped and Repetitive Behaviors and Interests, and (4) Social Initiation and Responsiveness. The Inattentive Behavior and Hyperactivity and Impulsivity domains were not rated, as newly funded projects have incorporated these two domains into a larger executive function-related behavior domain, with studies forthcoming.

During participants’ observation period, a study team member inspected the database at pre-selected time points (day 1, day 5, day 7, day 10, day 14) and monitored data logging. If a data point was missing, the day was assumed to be skipped, and the participant was contacted via e-mail or phone to encourage them to resume observation. If ratings were skipped, study personnel asked participants to compete additional days to obtain 14 complete ratings from each participant. At the conclusion of the observation, participants were asked to complete a set of validation measures, and a user feedback questionnaire.

### Measures.

The *Aberrant Behavior Checklist - Community* (ABC-C)^6^ is a global behavior checklist used to measure maladaptive behaviors among individuals with IDDs. The ABC-C consists of 58 items that target five behavioral dimensions (irritability, hyperactivity, lethargy/withdrawal, stereotypy, and inappropriate speech). Participants were instructed to complete the ABC-C based on behavior observed during the 2-weeks of iBehavior.

The *Behavior Rating Inventory of Executive Function 2* (BRIEF-2)^27^ is a 63-item proxy report of a child’s executive function and includes inhibition, shifting, emotional control, working memory, and planning and organization. Participants were instructed to complete the BRIEF-2 based on behavior observed during the 2-week period of iBehavior.

The *Conners 3 Short-Form*^10^ is an assessment of ADHD for children and adolescents ages 6 to 18 years. This 45-item assessment consists of the following subscales: inattention, hyperactivity/impulsivity, learning problems, executive functioning, aggression, and peer relations. Participants were instructed to complete the Connors 3 based on behavior observed during the 2-week observation period of iBehavior.

#### User Feedback.

All participants completed a 15-question user feedback survey that included questions about ease of use, technological problems, preference of iBehavior to other traditional questionnaires, as well as other aspects of usability, relevance, and satisfaction.

## Results

### Observation logging.

Participants completed observations and recorded iBehavior data on an average of 13.3 of 14 days (range = 12–14). In total, five participants did not skip a single observation. Three participants skipped their last one or two observations, and only two participants skipped a rating in the middle of their observation window, but resumed ratings the following day after an email or phone call reminder. Across the 140 possible observations, 8 were skipped, leading to a 94% response rate over 10 participants’ observation periods. Participants also completed 100% of items for each of their logged observations.

### iBehavior Scores.

To obtain domain summary scores, all items within their respective domain were summed over each observation day, generating a *total* domain score for each day. If participants indicated that the behavior in the item of interest was not present, a score of zero was assigned, thus, higher scores represented both greater frequency *and* intensity for the behavior, except for the social initiation and responsiveness domain, in which higher scores indicated *better* social initiation and responsiveness. Next, for each domain, total item scores for each day were summed, and then divided by the total number of observation days for each participant to account for differences in number of days observed across the sample. This procedure generated eight total scores, consisting of both frequency and intensity scores for each domain (means and standard deviations are reported in Table 1).

### Convergent Validity.

Due to the small sample size, Spearman rank-order correlations were conducted to assess associations between frequency and intensity ratings for each of the four domains of iBehavior, and validation measures (i.e., ABC-C, BRIEF-2, and Conners 3). For the ABC-C, the irritability sub-scale was significantly correlated with iBehavior irritable mood and aggression frequency (*r*(8) = .735, *p* = .015). Additionally, the ABC-C stereotypy domain was significantly related to iBehavior stereotyped and repetitive behaviors and interests intensity (*r*(8) = .742, *p* = .014). There were also associations with moderate correlation coeffi cients that did not reach statistical significance. For instance, on the ABC-C, the irritability sub-scale was related to iBehavior irritable mood and aggression intensity (*r*(8) = .613, *p* = .060), and on the ABC-C, the lethargy and social withdrawal subscale was negatively associated with both social initiation frequency (*r*(8) = − .517, *p* = .126) and intensity (*r*(8) = − .578, *p* = .080).

For the BRIEF-2, emotional control was associated with iBehavior irritable mood and aggression frequency (*r*(8) = .868, *p* = 001) and intensity (*r*(8) = .807, *p* = 005), as well as iBehavior avoidance, fearfulness and nervousness frequency (*r*(8) = .740, *p* = 014) and intensity (*r*(8) = .691, *p* = .027). In addition, BRIEF-2 shifting was associated with iBehavior stereotyped and repetitive behaviors frequency (*r*(8) = .817, *p* = .003) and intensity (*r*(8) = .835, *p* = .004).

### User Feedback.

**Figure 3:**
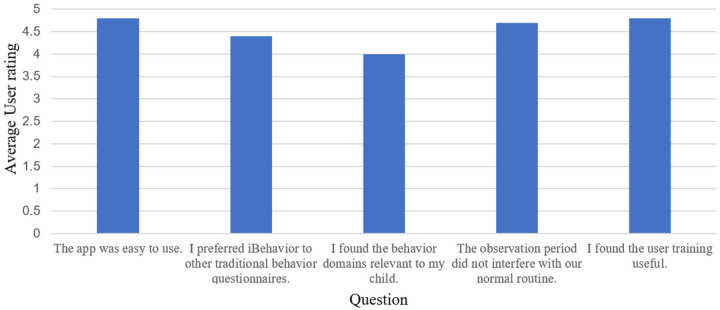
User feedback. Average user responses to the iBehavior feedback questionnaire, completed at the end of user’s 14-day observation period. Users rated the questions on a Likert scale with the following response options: Strongly disagree (1), disagree (2), neither disagree or agree (3), agree (4), and strongly agree (5).

Caregivers were positive about their experience using iBehavior. On a five-point Likert scale ranging from (1) strongly disagree to (5) strongly agree, participants generally indicated high satisfaction with the app (see Fig. 3). There were technical issues regarding text reminders being sent, and being sent at the correct times. These issues originated from errors in database set-up, and were resolved, however participants’ satisfaction with text reminders reflected these challenges (*m* = 2.5). See Fig. 3.

## Discussion

Results of the present study provide preliminary evidence that iBehavior is a feasible behavioral outcome measurement tool in IDDs, and may offer important advantages over traditional retrospective rating scales, in particular, due to its reliance on recording of behaviors shortly after they occur in context. We hypothesize that eEMA, compared to traditional rating scales, will reduce the contribution of expectancy bias to placebo responding^28–30^ in future controlled trials. We expect that this will occur because, instead of rating behaviors in the research clinic (for example at the end of each treatment period), caregivers will instead rate behaviors more objectively due to the proximity of time and place that the behaviors occur.

This hypothesis will be evaluated in an ongoing double blind, placebo-controlled crossover trial of liquid methylphenidate in 68 children and adolescents with IDD and comorbid ADHD (NCT05301361, Sensitivity of the NIH Toolbox to Stimulant Treatment in Intellectual Disabilities). If this and future trials confirm this hypothesis, several advances in clinical research may occur. First, reduced placebo responding and improved precision will increase statistical power to detect intervention benefits and reduce sample sizes needed for a desired effect size. Second, more frequent recording of key behaviors may help to reveal dynamic changes in behavior over time. Third, because user training includes discussion of each child’s unique behavior and how those behaviors are to be captured by the app, users may feel that their responses more accurately reflect their child, perhaps increasing accuracy and compliance.

This study was not without limitations. First, the small number of participants limited our power to detect relations between iBehavior domains and validation measures. Upcoming studies, including the aforementioned clinical trial and a larger feasibility study (n = 120) will be more adequately powered, particularly to assess reliability and validity. Second, all participants identified as biological mothers of the child they were observing, excluding fathers as reporters. Third, satisfaction and compliance of parents may be positively biased as those with interest in this technology or who feel negatively about traditional scales may be more likely to participate.

## Conclusion

Results of the present pilot study indicate successful implementation and preliminary feasibility and validity of an eEMA tool for use as a behavioral outcome measure in IDDs. We found evidence for convergent validity among our behavior domains and established caregiver report on the ABC-C, BRIEF-2 and Connors 3. Results from the user experience survey indicated satisfaction with the iBehavior app, and a preference to use it over traditional behavioral questionnaires. The creation of a secure smartphone-based eEMA measurement tool with targeted behavioral items specific to populations with IDDs is an innovative approach to detect treatment sensitivity, and serves as an important advancement in the field.

## Figures and Tables

**Table 1:** Means and Standard Deviations of iBehavior Domains

iBehavior Domain	Mean	SD
Aggression and Irritability Frequency	0.42	0.54
Aggression and Irritability Intensity	0.43	0.27
Restricted and Repetitive Behaviors Frequency	0.63	0.50
Restricted and Repetitive Behaviors Intensity	0.58	0.42
Avoidant and Fearful Frequency	0.16	0.16
Avoidant and Fearful Intensity	0.17	0.17
Social Initiation Frequency	1.97	0.77
Social Initiation Intensity	2.07	0.88
Hyperactivity	*Domain not included in the present study*	
Inattention	*Domain not included in the present study*	

## Data Availability

Data shall be made available upon request to the corresponding author.
